# Effects of Surgery on Survival of Early-Stage Patients With SCLC: Propensity Score Analysis and Nomogram Construction in SEER Database

**DOI:** 10.3389/fonc.2020.00626

**Published:** 2020-04-24

**Authors:** Yuyan Wang, Qiwen Zheng, Bo Jia, Tongtong An, Jun Zhao, Meina Wu, Minglei Zhuo, Jianjie Li, Jia Zhong, Hanxiao Chen, Xue Yang, Yujia Chi, Zhi Dong, Boris Sepesi, Jianjun Zhang, Carl M. Gay, Ziping Wang

**Affiliations:** ^1^Key Laboratory of Carcinogenesis and Translational Research (Ministry of Education/Beijing), Department of Thoracic Medical Oncology, Peking University Cancer Hospital & Institute, Beijing, China; ^2^Department of Epidemiology and Biostatistics, School of Public Health, Peking University, Beijing, China; ^3^Department of Thoracic and Cardiovascular Surgery, the University of Texas MD Anderson Cancer Center, Houston, TX, United States; ^4^Department of Thoracic/Head and Neck Medical Oncology, the University of Texas MD Anderson Cancer Center, Houston, TX, United States

**Keywords:** early-stage SCLC, surgery, prognosis, nomogram, propensity score analysis

## Abstract

**Purpose:** We aimed to assess the survival benefit of surgery for patients with stage IA–IIB small cell lung cancer (SCLC) and construct a nomogram for predicting overall survival (OS).

**Methods:** Patients who had been diagnosed with stage IA–IIB SCLC between 2004 and 2014 and who had received active treatment were selected from the Surveillance, Epidemiology, and End Results database. The primary endpoint was OS. Cox proportional hazards models and propensity score (PS) analyses were used to compare the associations between surgery and OS. The probability of 1- and 3-year OS was predicted using a nomogram.

**Results:** We reviewed 2,246 patients. The median OS of the surgery and non-surgery groups was 35 months and 19 months, respectively. Multivariable Cox proportional hazards models showed a survival benefit in the surgery group (hazards ratio [HR], 0.642; 95% confidence interval [CI], 0.557–0.740; *P* < 0.001). To balance the between-group measurable confounders, the impact of surgery on OS was assessed using PS matching. After PS matching, OS analysis still favored surgical resection. The PS-stratification, PS-weighting, and PS-adjustment models showed similar results to demonstrate a statistically significant benefit for surgery. Further, the nomogram was well calibrated and had good discriminative ability (Harrell's *C*-index = 0.645).

**Conclusion:** Our analysis suggests that surgery is a viable option for patients with early-stage SCLC. Our nomogram is a viable tool for quantifying treatment trade-off assumptions and may assist clinicians in decision-making. Future work is needed to validate our results and improve our tools.

## Introduction

The most commonly diagnosed cancer worldwide is lung cancer; it is the leading cause of cancer-related death worldwide, with 2018 recording ~2.1 million new cases and 1.8 million deaths (1). Small cell lung cancer (SCLC) is differentiated from non-SCLC (NSCLC) by its high growth fraction, rapid doubling time, and the early development of widespread metastases (2). SCLC involves 13~20% of all lung cancers. While its incidence in developed countries has declined in recent years, its frequency remains higher in developing countries, possibly due to variations in smoking practices and cigarette composition (3).

Most SCLC is sensitive to initial treatment, either platinum-based chemotherapy alone in advanced/metastatic disease, or platinum-based chemotherapy in combination with radiation in earlier stage disease, limited to a single hemithorax (i.e., limited stage). However, virtually all patients will eventually experience recurrent disease due to early dissemination and acquired drug resistance (4). At diagnosis, most patients with SCLC already have hematogenous metastases (i.e., extensive-stage disease). Consequently, locally advanced or distant metastatic disease (stage III/IV) will be diagnosed in >90% of patients (5), which contributes to the high mortality from the disease (6). Therefore, the 5-year survival rate remains low at <7% overall; most patients survive for only <1 year after diagnosis (7). There had been few improvements in SCLC standard of care management in the decades prior to the IMpower133 results, which demonstrated survival benefit with the addition of the anti–PD-L1 agent atezolizumab to platinum-based chemotherapy for patients with extensive-stage SCLC (8).

Surgical resection was once a mainstay of treatment for SCLC; however, survival outcomes were very poor. Subsequent studies demonstrating dramatic initial responses to chemotherapy and/or radiotherapy eventually shifted the SCLC standard of care away from surgical resection. Although previous research indicated the positive role of surgery in early-stage SCLC, the results remain controversial and inconsistent (9–12).

Therefore, we aimed to explore the value of surgery for the survival benefit of patients with stage IA–IIB SCLC and to construct a comprehensive, integrated nomogram to provide clinicians with a quantitative tool for estimating the overall survival (OS) of a patient with early-stage SCLC.

## Materials and Methods

### Study Participants

We obtained the study population from the Surveillance, Epidemiology, and End Results (SEER) program records of the National Cancer Institute. We selected patients diagnosed with SCLC as their original primary malignancy between 2004 and 2014 from the SEER database. The participants included patients with the following histologic codes (8002, 8041–8045) and site codes (C34.0–C34.3, C34.8–C34.9). We identified a total of 46,238 patients with SCLC in the SEER database. Only patients with early-stage SCLC (6th edition of American Joint Committee on Cancer [AJCC] staging I–II) were selected for further analysis. Patients were excluded if they had: (1) been diagnosed at autopsy or by death certificate only; (2) 990 or 996–999 tumor size code; (3) 950, 980, or 999 tumor extent code; and (4) lymph node involvement.

The surgery group comprised patients who received cancer-directed surgery. The non-surgery group comprised patients who did not receive cancer-directed surgery but who received radiotherapy and/or chemotherapy. We excluded patients with absence of active treatment codes (e.g., cancer-directed surgery, radiotherapy, or chemotherapy). We considered in our analysis 2,246 eligible patients who met our inclusion and exclusion criteria (Supplementary Figure 1).

The study was exempted from the Peking University Cancer Hospital Medical Ethics Committee. The data agreement was obtained and we downloaded the database directly from the SEER website in keeping with SEER requirements.

### Statistical Analysis

Categorical variables are reported as frequencies and proportions. Continuous variables are reported as means (standard deviation, SD) and medians (interquartile ranges, IQR). For comparing the baseline characteristics between the surgery and non-surgery groups, we used Student's *t*-test for normally distributed variables, the chi-square test or Fisher's exact test for categorical variables, and the Mann–Whitney *U*-test for all other continuous variables. We demonstrated and compared the crude survival differences between two groups using Kalan–Meier curves and log-rank tests. We examined the proportional hazards (PH) assumption and linearity assumption in continuous variables using restricted cubic splines (13, 14).

We transformed continuous variables to adequate form for fitting the PH and linearity assumptions. We assessed the impact of surgery on OS by Cox proportional hazard models with and without risk adjustment for age at diagnosis, sex, marital status, race, primary tumor location, anatomic sites, year of diagnosis, tumor grading, lymph node involvement, tumor size, AJCC stage, and extent of tumor. We estimated hazard ratios (HRs) and 95% confidence intervals (CI).

We performed propensity score (PS) analyses to reduce the potential confounders between the surgery and non-surgery groups (15–17). First, treatment assignment was predicted using multivariable logistic regression based on various confounders, including age at diagnosis, sex, marital status, ethnicity, laterality, anatomic sites, tumor grading, year of diagnosis, AJCC stage, tumor size, extent of tumor, and lymph node involvement. PS, which represented the probability of receiving surgery, was then calculated for each patient (18). Next, patients were matched 1:1 into surgery and non-surgery groups. We used the nearest neighbor method, with a logit SD caliper width of 0.2. The balances of matched covariates were measured by the standardized difference, and a difference between −0.1 and 0.1 was generally considered negligible (19). Additionally, three alternative approaches were used to adjust for the patients' non-random assignment: (1) Adjustment: The regression model included the PS as a covariate. (2) Weighting: Each patient was weighted by the PS by the inverse probability of being in the surgery versus non-surgery group with the aim of balancing the characteristics between the two groups (20). (3) Stratification: The patients were divided into five strata based on the quintile of the PS. The relationship between surgery and survival within each stratum was examined using Cox proportional hazards models; the five HRs of each stratum were joined into an overall HR using inverse variance weights under the fixed model (21, 22).

Based on the predictive model with the identified prognostic factors, we constructed a nomogram for predicting the 1- and 3-year OS probability. Backward stepwise selection with the Akaike information criterion (AIC) was used to select variables into the multivariate Cox proportional hazards regression model, and the predictors' coefficients were calculated. The performance of the nomogram included its discrimination; calibration was tested using internal validation. Discrimination is a model's ability to separate subject outcomes, indicated by Harrell's C index (23). We evaluated calibration, which compares predicted and actual survival, using a calibration plot. In a well-calibrated model, the predictions should fall on a 45-degree diagonal line. The goodness of fit was assessed using the Greenwood-Nam-D'Agostino goodness-of-fit test (24). The final reduced model-predicted probability of OS was compared with the observed OS at 1 and 3 years. The developed model underwent internal validation using bootstrapping technique based on 1,000 resamples.

All statistical analysis was performed with R software (version 3.3.3; http://www.r-project.org). We used the R packages “MatchIt” and “rms” were used for PS analysis and nomogram building. The reported significance levels were all two-sided; statistical significance was set at 0.05.

## Results

### Patient Characteristics and Factors Associated With Surgery

A total of 2,246 patients fulfilled the eligibility criteria. The median follow-up time was 23 months, and 1,467 deaths (65.3%) were observed. Table 1 presents the patients' baseline characteristics. The majority were female (53%), white (87%), and had N0 lymph node involvement (71%). The median age at diagnosis was 68 years (IQR, 61–75 years). The median tumor size was 3.0 cm (IQR, 2.0–4.6 cm). Overall, 618 patients (27.5%) had cancer-directed surgery and 1,628 patients (72.5%) received radiotherapy and/or chemotherapy without surgical resection. In line with previous studies, the patients without cancer-directed surgery were elderly, non-white, with larger tumor sizes, and higher N stages.

**Table 1:** Comparison of baseline characteristics between surgery and non-surgery groups among patients with stage I–II SCLC in the original and matched data sets.

**Characteristics**	**Original cohort**								**Matched cohort**						
	**Total (*****N*** **=** **2246)**		**Surgery (*****N*** **=** **618)**		**Non-surgery (*****N*** **=** **1628)**		***P*-value**	**Sdiff**	**Total (*****N*** **=** **950)**		**Surgery (*****N*** **=** **475)**		**Non-surgery (*****N*** **=** **475)**		**Sdiff**
Age at diagnosis, years															
Mean (SD)	67.5	(9.8)	66.7	(9.4)	67.8	(10)	0.006	−0.118	67.0	(9.3)	67.1	(9.3)	66.8	(9.3)	0.029
Median (IQR)	68	(61–75)	67	(60–74)	69	(61–75)			67	(61–74)	67	(60–74)	68	(61–73)	
Sex															
Female	1,190	(53.0)	344	(55.7)	846	(52.0)	0.128	0.074	526	(55.4)	261	(54.9)	265	(55.8)	−0.017
Male	1,056	(47.0)	274	(44.3)	782	(48.0)		−0.074	424	(44.6)	214	(45.1)	210	(44.2)	0.017
Race															
White	1,957	(87.1)	575	(93.0)	1,382	(84.9)	<0.001	0.258	872	(91.8)	439	(92.4)	433	(91.2)	0.046
Black	198	(8.8)	30	(4.9)	168	(10.3)		−0.205	53	(5.6)	25	(5.3)	28	(5.9)	−0.028
Asian/Other	91	(4.1)	13	(2.1)	78	(4.8)		−0.147	25	(2.6)	11	(2.3)	14	(2.9)	−0.039
Marital status															
Married	1,165	(51.9)	339	(54.9)	826	(50.7)	0.09	0.082	495	(52.1)	257	(54.1)	238	(50.1)	0.08
Unmarried	1,081	(48.1)	279	(45.1)	802	(49.3)		−0.082	455	(47.9)	218	(45.9)	237	(49.9)	−0.08
Anatomic site															
Upper	1,278	(56.9)	368	(59.5)	910	(55.9)	<0.001	0.074	552	(58.1)	274	(57.7)	278	(58.5)	−0.017
Middle	131	(5.8)	42	(6.8)	89	(5.5)		0.055	63	(6.6)	33	(6.9)	30	(6.3)	0.025
Lower	635	(28.3)	190	(30.7)	445	(27.3)		0.075	293	(30.8)	151	(31.8)	142	(29.9)	0.041
Bronchus/Other	202	(9.0)	18	(2.9)	184	(11.3)		−0.322	42	(4.4)	17	(3.6)	25	(5.3)	−0.082
Laterality															
Left-sided	1,008	(44.9)	274	(44.3)	734	(45.1)	0.786	−0.015	428	(45.1)	211	(44.4)	217	(45.7)	−0.025
Right-sided	1,238	(55.1)	344	(55.7)	894	(54.9)		0.015	522	(54.9)	264	(55.6)	258	(54.3)	0.025
Tumor grading															
Well or moderate	40	(1.8)	27	(4.4)	13	(0.8)	<0.001	0.224	26	(2.7)	14	(2.9)	12	(2.5)	0.026
Poor	407	(18.1)	215	(34.8)	192	(11.8)		0.525	240	(25.3)	122	(25.7)	118	(24.8)	0.019
Undifferentiated	556	(24.8)	184	(29.8)	372	(22.9)		0.157	321	(33.8)	151	(31.8)	170	(35.8)	−0.084
NOS	1,243	(55.3)	192	(31.1)	1,051	(64.6)		−0.636	363	(38.2)	188	(39.6)	175	(36.8)	0.056
Year of diagnosis															
2004–2007	858	(38.2)	237	(38.3)	621	(38.1)	0.722	0.004	371	(39.1)	185	(38.9)	186	(39.2)	−0.004
2008–2011	781	(34.8)	221	(35.8)	560	(34.4)		0.029	327	(34.4)	168	(35.4)	159	(33.5)	0.04
2012–2014	607	(27.0)	160	(25.9)	447	(27.5)		−0.035	252	(26.5)	122	(25.7)	130	(27.4)	−0.038
AJCC 6^th^ stage															
IA	699	(31.1)	285	(46.1)	414	(25.4)	<0.001	0.422	409	(43.1)	202	(42.5)	207	(43.6)	−0.021
IB	812	(36.2)	172	(27.8)	640	(39.3)		−0.241	271	(28.5)	138	(29.1)	133	(28.0)	0.023
IIA	279	(12.4)	76	(12.3)	203	(12.5)		−0.005	122	(12.8)	63	(13.3)	59	(12.4)	0.025
IIB	456	(20.3)	85	(13.8)	371	(22.8)		−0.232	148	(15.6)	72	(15.2)	76	(16.0)	−0.023
Tumor size, cm															
Mean (SD)	3.6	(2.2)	2.5	(1.5)	4.0	(2.3)	<0.001	−0.769	2.8	(1.6)	2.8	(1.6)	2.9	(1.7)	−0.067
Median (IQR)	3.0	(2.0–4.6)	2.1	(1.5–3.0)	3.5	(2.3–5.0)			2.5	(1.7–3.5)	2.5	(1.7–3.5)	2.5	(1.8–3.5)	
Extent of tumor															
Local	1,780	(79.3)	466	(75.4)	1,314	(80.7)	0.007	−0.128	737	(77.6)	368	(77.5)	369	(77.7)	−0.005
Regional	466	(20.7)	152	(24.6)	314	(19.3)		0.128	213	(22.4)	107	(22.5)	106	(22.3)	0.005
Lymph node involvement															
N0	1,601	(71.3)	475	(76.9)	1,126	(69.2)	<0.001	0.173	716	(75.4)	357	(75.2)	359	(75.6)	−0.010
N1	645	(28.7)	143	(23.1)	502	(30.8)		−0.173	234	(24.6)	118	(24.8)	116	(24.4)	0.010

*AJCC, American Joint Committee on Cancer; Sdiff, standardized difference; SD, standard deviation; IQR: interquartile range*.

### Survival Analysis

**Figure 1:**
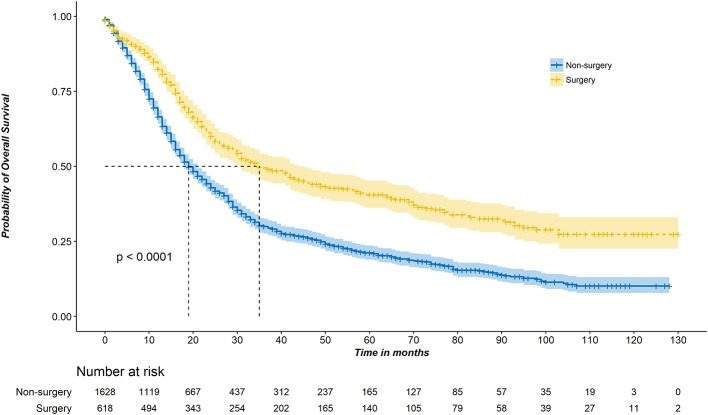
Kaplan–Meier survival curves for IA–IIB stage SCLC patients with or without surgery.

Figure 1 shows the Kaplan–Meier survival curve. The median OS of the surgery group was 35 months (95% CI, 31–44 months) compared with 19 months (95% CI, 18–21 months) in the non-surgery group (*P* < 0.001). The unadjusted 3-year survival probability was 30.0% (95% CI, 27.6–32.6%) for patients in the non-surgery groups vs. 49.4% (95% CI, 45.3–54.0%) for those in the surgery groups. Controlling for demographic and clinical characteristics in the multivariable Cox regression model revealed a significant difference in OS between the surgery and non-surgery patients (HR, 0.642; 95% CI, 0.557–0.740; *P* < 0.001) (Table 2).

**Table 2:** Effect of surgery on hazard ratios for overall survival.

**Models**	**Sample size, No**.			**HR (95% CI)**	
	**Surgery**	**Non-surgery**	**Events**	**HR (95% CI)**	***P* value**
Unadjusted model	618	1,628	1467	0.572 (0.506–0.647)	<0.001
Multivariable-adjusted model ^a^	618	1,628	1467	0.642 (0.557–0.740)	<0.001
Propensity score-adjusted model ^b^					
Within-propensity score quintile					
Quintile 1 (Lowest propensity score)	23	427	324	0.792 (0.457–1.372)	0.405
Quintile 2	42	407	302	0.495 (0.316–0.776)	0.002
Quintile 3	86	363	300	0.614 (0.446–0.845)	0.002
Quintile 4	160	290	293	0.782 (0.606–1.010)	0.060
Quintile 5 (Highest propensity score)	307	141	248	0.525 (0.395–0.696)	<0.001
Combined ^c^	618	1,628	1,467	0.635 (0.548–0.736)	<0.001
Regression adjustment	618	1,628	1,467	0.649 (0.561–0.749)	<0.001
Weighting (IPTW)	618	1,628	1,467	0.673 (0.597–0.759)	<0.001
Matching 1:1	475	475	598	0.690 (0.585–0.814)	<0.001

HR, hazard ratio; CI, confidence interval; IPTW, inverse probability of treatment weighting.

a Adjusted for age, sex, ethnicity, marriage status, anatomic sites, laterality, tumor grading, year of diagnosis, AJCC stage, tumor size, extent of tumor, and lymph node involvement.

b The propensity of receiving surgery was estimated using a multivariable logistic regression model that included baseline age, sex, race, marital status, anatomic sites, laterality, tumor grading, year of diagnosis, AJCC stage, tumor size, extent of tumor and lymph node involvement.

c *Results were combined among strata using inverse variance weights under a fixed model, as there was no detectable heterogeneity among strata (all P for heterogeneity = 0.2)*.

PS analyses were performed to balance measurable confounders between the groups and to evaluate whether surgery has a favorable prognostic impact on survival. We stratified patients into quintiles based on the PS, and assessed the effect of surgery on survival. In each of the five strata, the surgical patients had better outcome compared with non-surgical patients (Supplementary Figure 2). As there was no detectable heterogeneity across strata, we used inverse variance weights under the fixed model to combine the five HRs estimated from each stratum, and obtained the overall HR for the whole cohort (HR, 0.635; 95% CI, 0.548–0.736; *P* < 0.001) (Table 2). Apart from PS stratification method, we also used other alternative methods, including matching, weighting, and regression adjustment.

Before matching, the surgical patients had a PS of 0.482 ± 0.250 compared to 0.178 ± 0.250 in the non-surgery group (*P* < 0.001), indicating a strong and clinically relevant bias regarding the two groups' observed demographic and clinical characteristics. When PS matching was used, the surgery and non-surgery groups were well-matched (475 patients each), with no significant differences in clinical and pathologic factors (Table 1, Supplementary Figure 3). Among the PS-matched pairs, surgical patients showed better OS (3-year OS rate, 47.6%) than non-surgical patients (3-year OS rate, 33.9%; Supplementary Figure 4). The multivariable Cox proportional hazards models also showed a significant benefit in OS for the surgery group (HR, 0.690; 95% CI, 0.585-0.814; *P* < 0.001) (Table 2). Inverse probability weighted Cox models or PS adjustment strategy also showed consistent results that demonstrated favorable evidence supporting the advantage of surgery treatment in patients with stage IA–IIB SCLC (Table 2).

**Figure 2:**
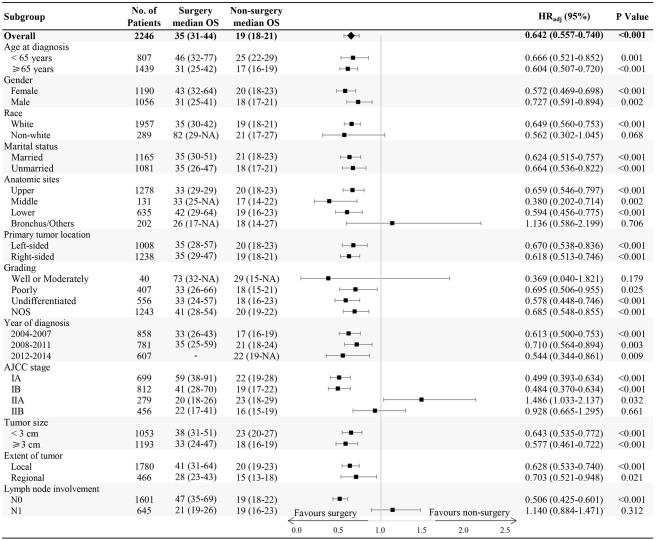
Forest plot of the subgroup analysis.

In subgroup analysis, OS benefit was observed across all subgroups in surgical patients compared with those in the non-surgery group, except for non-white race, bronchus and other anatomic sites, stage IIA or IIB, well or moderately differentiated grading, and N1 lymph node involvement (Figure 2).

### Nomogram and Model Performance

Restricted cubic splines were used to explore the effects of continuous variables. Tumor size and age had nonlinear effects on the log of HR of OS (Supplementary Figure 5). Seven variables that were the most associated with OS were identified based on backward stepwise procedure: tumor size, age, extent of tumor, N stage, surgery, radiotherapy, and chemotherapy. In addition, significant interactions were noted between surgery and radiotherapy, and surgery and N stage; that is, the effect size of surgery varied by nodal status and radiotherapy treatment (*P* < 0.001, Supplementary Table 1).

**Figure 3:**
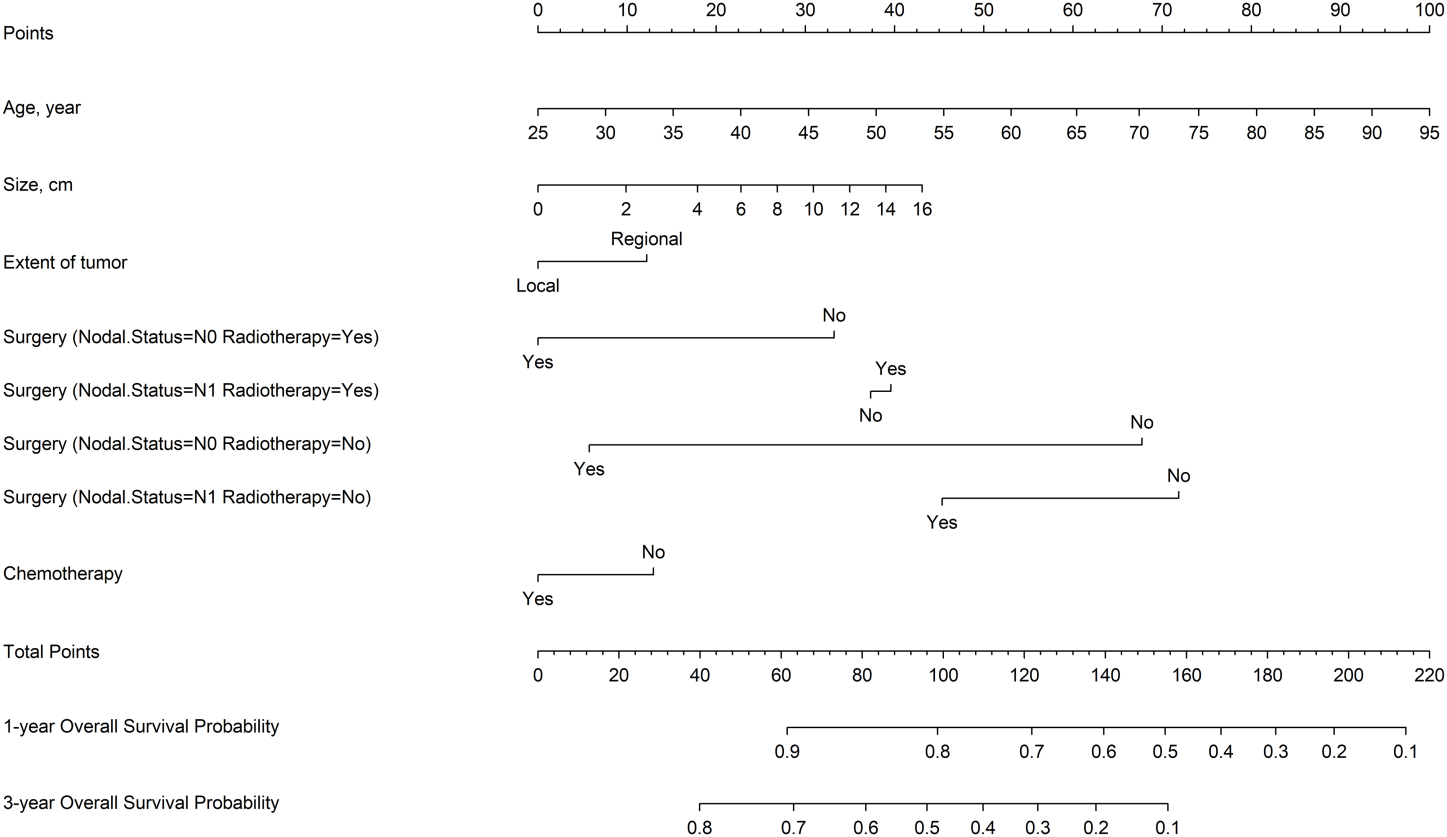
Nomogram for predicting OS of patients with IA–IIB SCLC.

**Figure 4:**
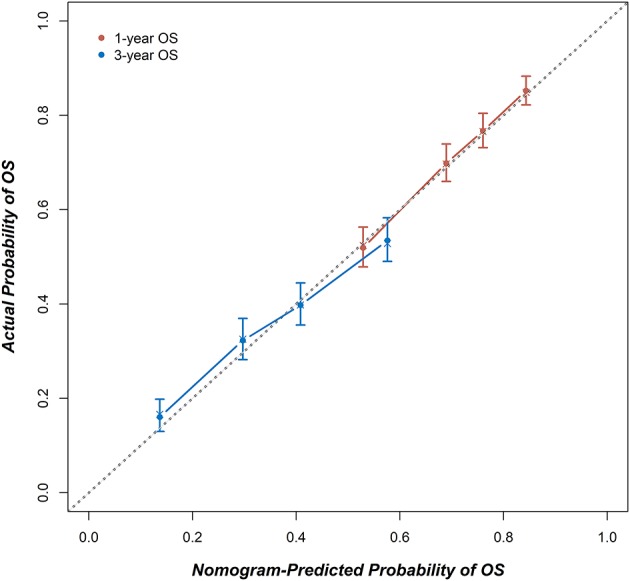
The calibration curves for predicting 1- and 3-year OS. X-axis: The OS probability predicted by the nomogram; y-axis: the actual OS probability. A plot along the 45-degree dotted line indicates a perfect calibration model, where the predicted probabilities and actual outcomes are identical.

Figure 3 shows a nomogram model for predicting the 1- and 3-year OS of patients with stage IA–IIB SCLC with active treatment. The Harrell's *C*-index for the established nomogram (0.645; 95% CI, 0.630–0.662) was significantly higher than the tumor-node-metastasis (TNM) staging system (0.549; 95% CI, 0.529–0.565; *P* < 0.001). The Hosmer-Lemeshow goodness-of-fit test showed that the nomogram had adequate calibration (*P* = 0.728). Figure 4 presents the nomogram's 1000-sample bootstrapped calibration plot for predicting the 1- and 3-year OS. The calibration plots revealed good predictive accuracy of the nomogram.

## Discussion

In this retrospective cohort study, we compared, via the SEER database, the survival outcomes for patients with early-stage SCLC who were treated with or without cancer-directed surgery. By using multivariable regression and PS analyses to reduce confounding, our results consistently demonstrate that treatment with surgery is associated with statistically significantly improved median OS in patients with stage I and II SCLC. Additionally, we present a nomogram for estimating the OS for patients with early-stage SCLC. It can be used to quantify assumptions about treatment trade-off and to guide the management of patients with SCLC.

Although NSCLC remains the most common lung cancer, SCLC nevertheless constitutes a major percentage of advanced-stage lung cancers (25). With the advances in chemotherapy and radiation treatment modalities, surgical resection of even early-stage SCLC tumors had fallen out of favor. However, several recent studies have suggested that, for a select group of patients with early-stage disease, surgery may be a reasonable, and in fact, superior SCLC treatment modality. Unfortunately, these data were based primarily on retrospective analyses, and few prospective data are available on which to base the consideration for surgical resection. Previous retrospective studies have found patients with stage I and II SCLC have prolonged survival following surgical resection as compared to non-surgical treatment models (26–31).

In the present study, we found that treatment with surgery was associated with statistically significantly improved median survival among patients with early-stage SCLC. Stratified analysis showed OS benefit across all subgroups in the surgical patients compared with non-surgery group, with the following exceptions: non-white race, well or moderately differentiated grading, stage IIA or IIB, bronchus and other anatomic sites, and N1 lymph node involvement. The number of patients reviewed who were non-white (289/2246), had bronchus and other anatomic site involvement (202/2246), and well or moderately differentiated grading (40/2246) was relatively low. Considering the relatively small sample size, the result is not statistically significant (*P* > 0.05). It is possible that among non-white patients with SCLC, cultural differences underlie patient preferences for (or against) surgical resection. As for tumors located in or invading the bronchus, this anatomical site may increase the difficulty of the surgical approach, which may limit R0 resections and lead to increased post-operative complications. Histologically, SCLC is usually poor or undifferentiated; while in the present study 40 patients were diagnosed as having well or moderately differentiated grading. The pathological characteristic might identify patients who respond better to chemotherapy or radiotherapy and lower malignant/metastatic potential. The present study included patients with stage IIA or IIB disease; we found the survival was not significantly prolonged in surgical group compared with those with stage I disease. N1 lymph node involvement is also an indicator of decreased benefit from surgical resection. Although the total population showed survival benefit in the surgery group, subgroup analysis showed that patients with stage II and N1 lymph node metastasis did not benefit from surgery. This result confirmed that surgery should be included in popular multimodality treatment for patients with stage I SCLC as one of the most important therapy.

Multivariate Cox proportional hazards regression analysis showed that with or without PS analysis, the HR still showed benefit in the surgery group. While other studies have suggested that the poor efficacy of current treatment options for disease progression is related to the lack of benefit of early diagnosis, as such subjects do not usually have the option of surgery, as systemic spread and paraneoplastic syndrome typically impede the therapeutic potential of resection (32). Improved or favorable survival rates in patients with resected SCLC have been described in recent retrospective analyses (33, 34), indicating that surgery is associated with improved survival for certain patients with early-stage SCLC (35). The results of the present study are almost identical to that of these previous studies (36).

Nowadays, SCLC in 80–90% of cases is still being diagnosed as TNM stage III–IV (37), and the Staging and Prognostic Factors Committee Advisory Boards and Participating Institutions have confirmed the prognostic value of clinical and pathological TNM staging in patients with SCLC and recommend continued usage for SCLC in the 8th edition of the TNM classification for lung cancer (37, 38). In our nomogram, seven variables were identified as most closely associated with OS: age, chemotherapy, extent of tumor, tumor size, surgery, N stage, and radiotherapy. Additionally, significant interactions were observed between surgery and radiotherapy/N stage, which demonstrated that the effect of the surgery modality varied with different nodal status as well as radiotherapy treatment. The established nomogram model for predicting 1- and 3-year OS of patients with IA–IIB SCLC had a significantly higher Harrell's *C*-index than the TNM staging system. This indicates that only TNM staging alone is insufficient for defining the prognosis of patients with early-stage SCLC. Further studies with external samples are needed to validate our predictive model.

Our study has several apparent limitations that need to be addressed. First, the SEER database lacks essential clinical details, such as baseline lung function, specific chemotherapy regimen, the treatment sequence following disease progression, information on gene mutation, and tobacco usage, which may be associated with surgery selection, survival, or both. Our estimation of the effect of surgery might be biased by these unmeasurable potential confounders. The results therefore should be interpreted with caution. Second, our ability to identify the patients who would benefit more from treatment with surgery was limited by the comparatively small sample, especially in subgroup analysis. Third, internal validation was used to evaluate model performance via the bootstrap approach. Although good performance was demonstrated, we still require independent cohort–based external validation to assess the accuracy of the model.

In summary, our study demonstrates that treatment with surgery is associated with statistically significantly improved median OS among patients with early-stage SCLC. We have also established a nomogram for predicting the 1- and 3-year OS probability. Our nomogram showed relatively good performance and could become useful personalized predictive tool for prognosis in SCLC patients. Future work with more detailed clinical information might improve our tool, and our results need to validate with large-scale prospective cohorts.

## Data Availability Statement

Publicly available datasets were analyzed in this study. This data can be found here: Data are available from www.seer.cancer.gov.

## Ethics Statement

The studies involving human participants were reviewed and approved by Medical Ethics Committee of Peking University Cancer Hospital. The patients/participants provided their written informed consent to participate in this study.

## Author Contributions

ZW and CG conceived the study, undertook project leadership and are guarantors of this work. YW and QZ wrote the first draft of the manuscript. QZ, YW, and BJ analyzed data and interpreted the data. TA, JZha, MW, MZ, JL, JZho, HC, XY, YC, ZD, BS, and JZhan were responsible for data acquisition and interpretation. All authors contributed to the drafting and critical revision of the manuscript. All authors approved the final version of the manuscript.

## Conflict of Interest

The authors declare that the research was conducted in the absence of any commercial or financial relationships that could be construed as a potential conflict of interest.

## Abbreviations

SCLC: small cell lung cancer
NSCLC: non-small-cell lung carcinoma
OS: overall survival
SEER: Surveillance, Epidemiology, and End Results
AJCC: American Joint Committee on Cancer
PH: proportional hazard
HR: hazard ratio
CI: confidence interval
PS: propensity score
IPTW: inverse probability of treatment weighting
AIC: Akaike information criterion
IQR: interquartile range
TNM: tumor-node-metastasis.
