# Supplementary material for: Effects of Surgery on Survival of Early-Stage Patients With SCLC: Propensity Score Analysis and Nomogram Construction in SEER Database
Source: Front Oncol. 2020 Apr 24;10:626. doi: 10.3389/fonc.2020.00626 (PMC7193096; doi:10.3389/fonc.2020.00626)
Supplement: Supplementary file 1 [file Table_1.DOCX]

**Figure S1.** Flow chart of study patients’ selection.

**
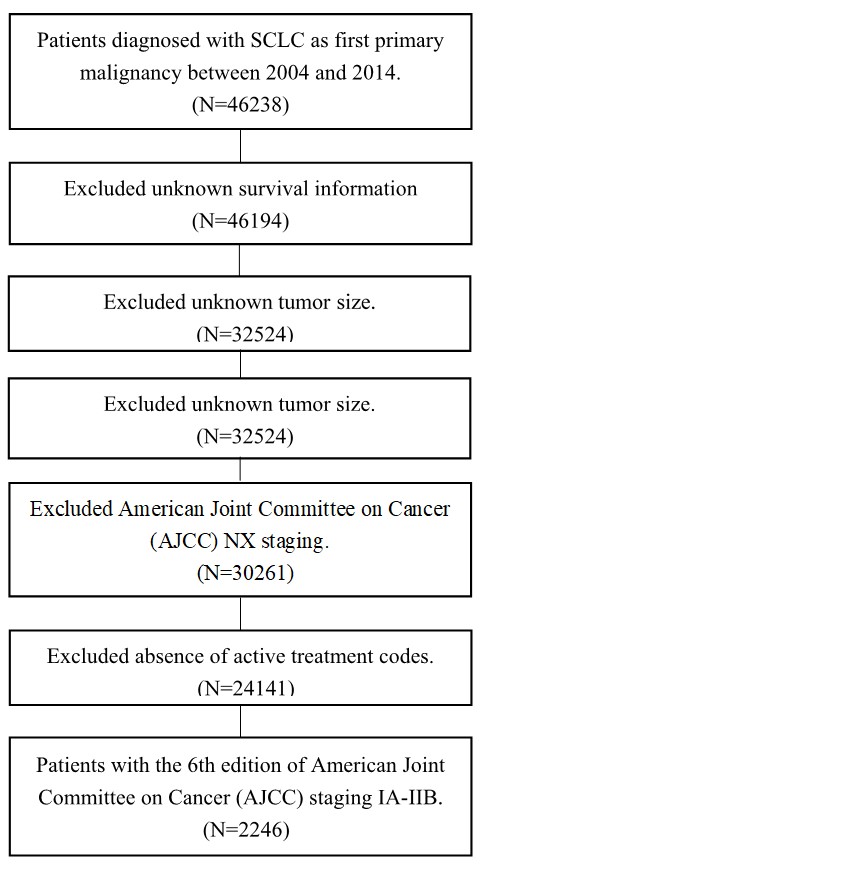
**

**Figure S2.** Kaplan-Meier survival curves for I-II stage SCLC patients with or without surgery across five propensity score strata. A. propensity score quintile 1 group; B. propensity score quintile 2 group; C. propensity score quintile 3 group; D. propensity score quintile 4 group; and E. propensity score quintile 5 group.

**
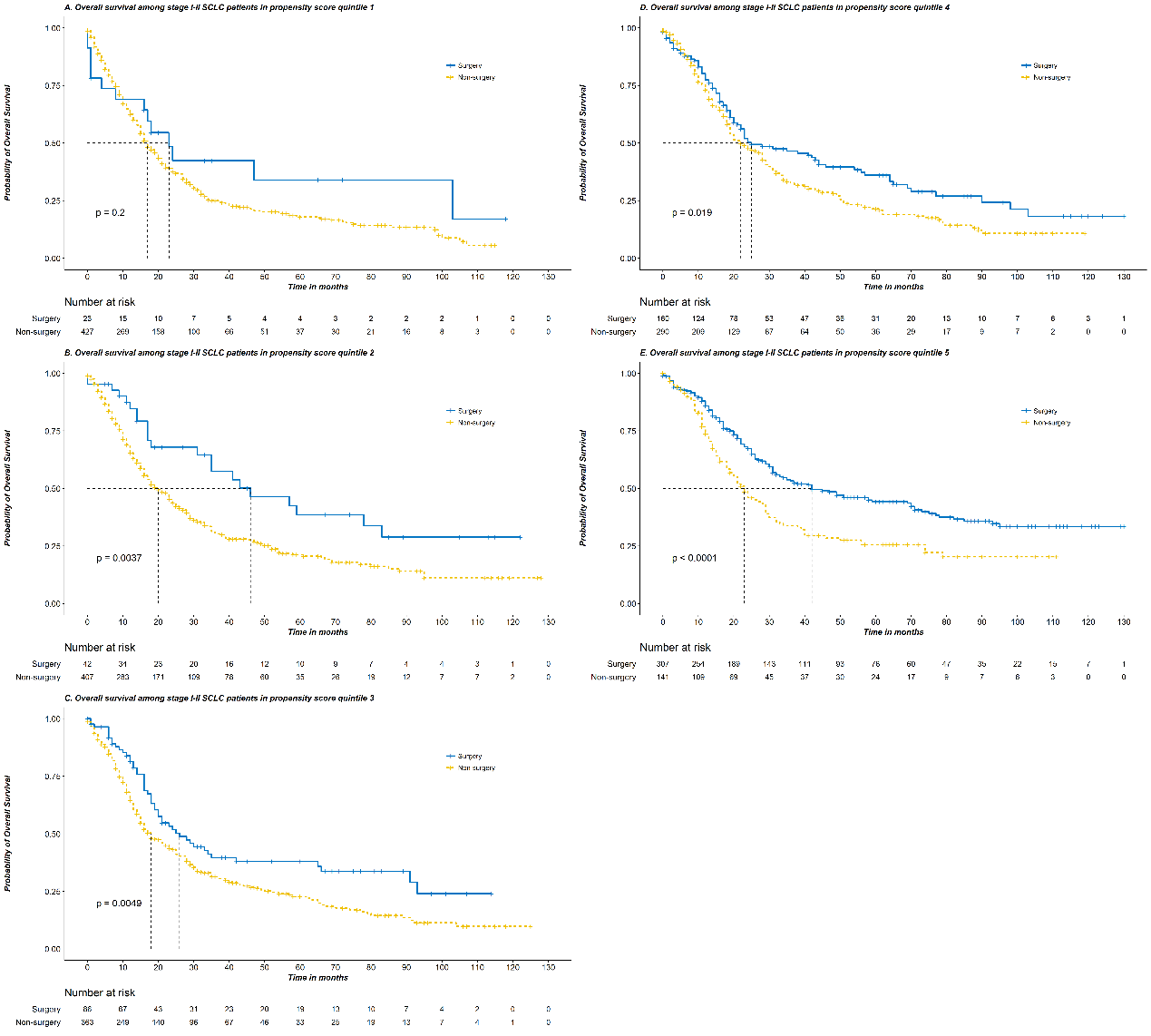
**

**Figure S3.** Propensity score distributions (**A**) before matching, and (**B**) after matching.


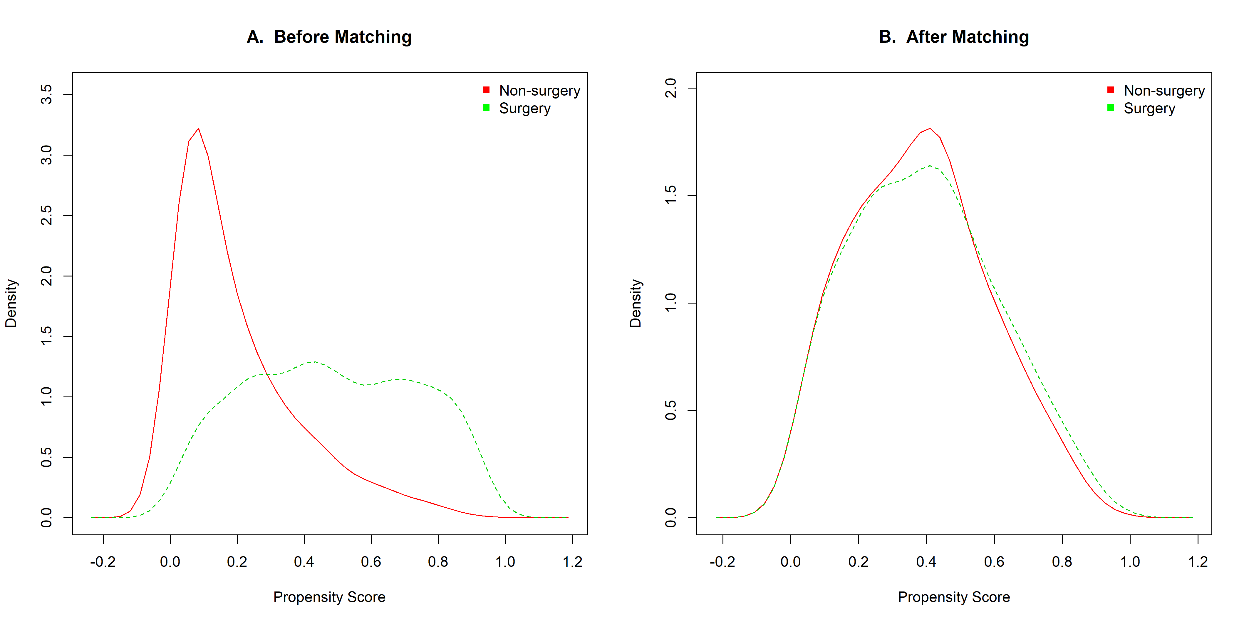


**Figure S4.** Kaplan-Meier survival curves for I-II stage SCLC patients with or without surgery after propensity score matching.


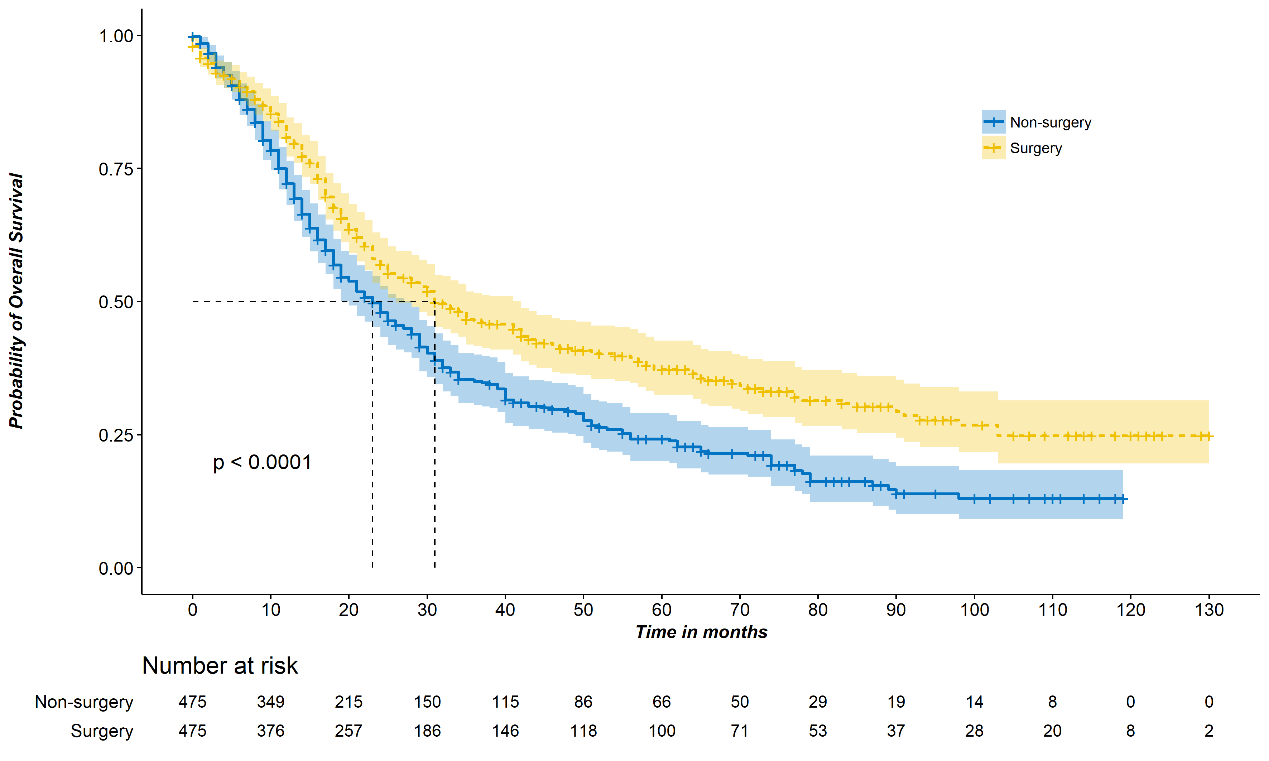


**Figure S5.** Transformation of Continuous Variables in Univariate Analysis Using Restricted Cubic Splines relating to (**A**) age and (**B**) tumor size.

**
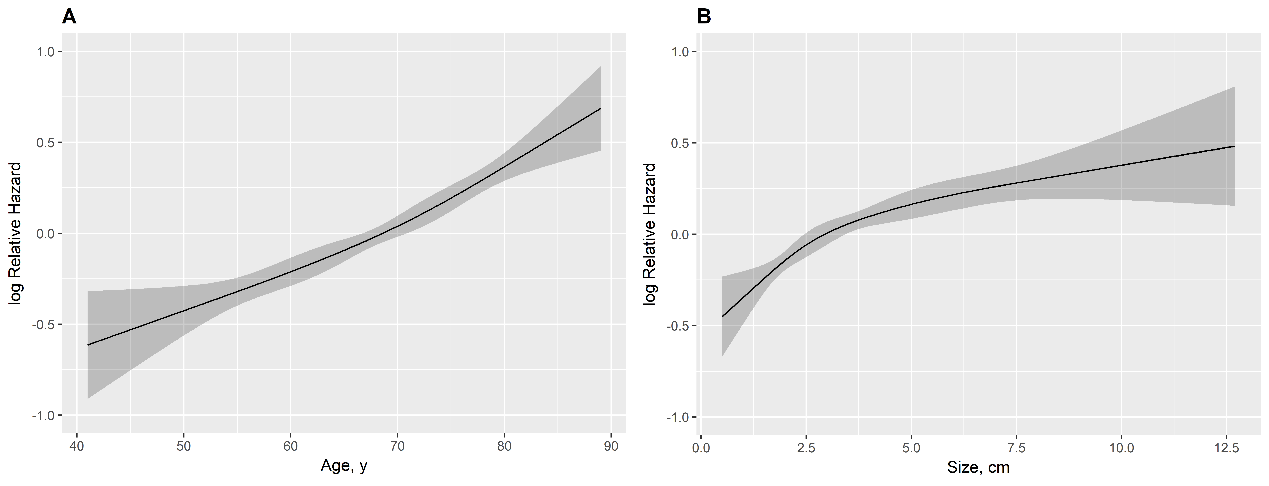
**

**Table S1.** Cox proportional hazard multivariable regression model parameters.

| Covariate | Beta coefficient | P |
| --- | --- | --- |
| Age ^a^ | 0.031 | <0.001 |
| Age’ | -0.003 | 0.692 |
| Tumor size ^b^ | 0.100 | 0.012 |
| Tumor size’ | -0.064 | 0.264 |
| Regional extension | 0.246 | <0.001 |
| N1 stage | 0.083 | 0.208 |
| Surgery | -1.252 | <0.001 |
| Chemotherapy | -0.261 | 0.002 |
| Radiotherapy | -0.697 | <0.001 |
| Surgery : Radiotherapy | 0.581 | <0.001 |
| Surgery : N1 stage | 0.716 | <0.001 |

^a^ Age modeled using restricted cubic spline function with three knots, requiring two independent coefficients: age and age’.

^b^ Tumor size modeled using restricted cubic spline function with three knots, requiring two independent coefficients: tumor size and tumor size’.
